# Environmental exposures at home and in the workplace in relation to inhaled corticosteroid medication - the population-based REGAL study

**DOI:** 10.1186/s12890-026-04502-w

**Published:** 2026-07-20

**Authors:** Juan Wang, Andreas Palm, Caroline Ahlroth Pind, Mathias Holm, Sven-Erik Dahlén, Lars Modig, Ane Johannessen, Xingwu Zhou, Andrei Malinovschi, Össur Ingi Emilsson

**Affiliations:** 1 Department of Medical Sciences, Respiratory-, Allergy- and Sleep Research, Uppsala University, Uppsala, SE-751 85 Sweden; 2 Department of Medical Sciences, Clinical Physiology, Uppsala University, Uppsala, Sweden; 3 Centre for Clinical Research Västmanland, Uppsala University, Västerås, Sweden; 4 Occupational and Environmental Medicine, School of Public Health and Community Medicine, Institute of Medicine, University of Gothenburg, Gothenburg, Sweden; 5 Clinical Lung and Allergy Research Unit, Department of Medicine Huddinge, Karolinska Institutet, Stockholm, Sweden; 6 Department of Respiratory Medicine and Allergy, Karolinska University Hospital, Stockholm, Sweden; 7 Unit of Integrative Metabolomics, Institute of Environmental Medicine, Karolinska Institutet, Stockholm, Sweden; 8 Department of Epidemiology and Global Health, Umeå University, Umeå, Sweden; 9 Department of Global Public Health and Primary Care, University of Bergen, Bergen, Norway; 10 Faculty of Medicine, University of Iceland, Reykjavik, Iceland; 11 Department of Respiratory Medicine, Landspitali University Hospital, Reykjavik, Iceland

**Keywords:** Indoor dampness, Irritating air, Traffic-related air pollution, Occupational exposure to fumes, ICS prescription

## Abstract

**Background:**

Diverse environmental exposures — home, occupational, and traffic-related — may impact asthma, but population-based studies on objectively assessed asthma are lacking. We aimed to investigate the impact of these exposures on future inhaled corticosteroid (ICS) use.

**Methods:**

Data from two Swedish population-based cohorts (performed in 2008–2010) were linked to the National Prescribed Drug Registry from 2005 to 2018 to form the REGAL study. This analysis included 31,768 participants from Stockholm, Gothenburg, Uppsala, and Umeå. Participants reported asthma diagnosis, asthma-related symptoms, and home and occupational exposures via questionnaires, while ICS prescriptions were retrieved from registry.

**Results:**

Among 31,768 participants, 6.9% (*n* = 2,103) reported asthma medication via questionnaire at baseline, and 5.3% (*n* = 1,679) initiated ICS therapy during the follow-up period (median follow-up time: 10.7 years). The prevalence of floor dampness, irritating air at home, traffic exposure at home, and occupational exposure to gas, smoke, or dust were 3.8%, 17.9%, 4.0%, and 36.7%, respectively. Floor dampness (odds ratio OR = 1.32, 95%CI 1.06–1.63), irritating air (OR = 2.08, 95%CI 1.85–2.33), traffic exposure at home (OR = 1.48, 95%CI 1.21–1.81), and occupational exposure to gas, smoke, or dust (OR = 1.55, 95%CI 1.39–1.72) were associated with self-reported asthma medication. An increased risk of initiating ICS therapy during the follow-up period was found for floor dampness at home (hazard ratio HR = 1.42, 95%CI 1.14–1.77), irritating air at home (HR = 1.46, 95%CI 1.29–1.65), and previous occupational exposure to gas, smoke, or dust (HR = 1.25, 95%CI 1.12–1.40).

**Conclusions:**

Indoor dampness, irritating air at home, and occupational exposure to gas/smoke/dust were associated with self-reported asthma medication at baseline and future initiation of ICS.

**Supplementary Information:**

The online version contains supplementary material available at 10.1186/s12890-026-04502-w.

## Background

Indoor dampness and mould are commonly linked to biological contaminants such as bacteria and house dust mites [1–3], and can contribute to the release of microbial volatile organic compounds and degradation-related chemicals from building materials [3, 4]. A previous review identified exposure to residential dampness and mould as a risk factor for the development of asthma [4]. Growth of bacteria or mould in indoor environments can lead to irritated air such as mould odour. Among various indicators of indoor dampness, mould odour showed the strongest association with asthma onset [4]. A recent review evaluating the effects of major indoor air pollutants—such as cleaning agents, dampness and mould, and volatile organic compounds—on the risk of new-onset asthma and related outcomes found moderate-certainty evidence only for an association between indoor mould exposure and new-onset asthma [5]. Only a few of the studies included in these two reviews specifically addressed adult-onset asthma [6, 7]. More recently, two prospective studies from Europe have provided additional evidence linking household dampness and mould to the development of asthma and respiratory symptoms in adults [8, 9]. However, these studies did not evaluate whether these associations had implications for the need for asthma treatment, which would have further strengthened their clinical relevance.

Traffic-related air pollution has been linked to onset of asthma in children [10, 11], however, the evidence regarding its effects on adult-onset asthma is limited [12, 13]. Three population-based cohort studies have linked residential traffic-related air pollution to asthma onset. A Swiss study with a 10-year follow-up period reported that higher particulate matter exposure increased incidence among never-smokers [14], while a Danish cohort found that long-term nitrogen dioxide (NO_2_) exposure raised the risk of first hospital admission for asthma in older adults [15]. A pooled analysis of cohorts from Denmark and Sweden further showed that exposure to particulate matter, NO₂, and black carbon at home address was associated with adult asthma incidence [16].

There has been growing recognition of the importance of adult-onset asthma induced by occupational exposure to irritants and cleaning agents [17]. Reviews have demonstrated associations between occupational exposure to organic or wood dust and asthma risk [18, 19], as well as increased asthma risk among nurses exposed to cleaning and disinfecting agents [20]. However, few population-based prospective studies on this topic are available [21–26]. Longitudinal European cohorts have linked irritants such as chemical spills, wood dust, vapours, gases, dust, and fumes, disinfectants or cleaning products to an increased risk of incident asthma [21–25]. None of these studies evaluated the use of asthma medication or examined other possible asthma-related exposures.

Therefore, there is a lack of population-based studies examining a broad range of environmental exposures—including the home environment, occupational exposure, and traffic-related exposure—in relation to the development of asthma. In this study, data from two Swedish population-based cohorts were linked to national drug register, collectively forming the REGAL study. The first aim was to investigate indoor dampness and mould exposure, perceived air irritation, traffic-related exposure at home, and occupational exposure to gas, smoke, or dust in relation to self-reported asthma medication. The second aim was to study the effects of these exposures on subsequent initiation of inhaled corticosteroid (ICS) therapy.

## Methods

### Study population

**Fig. 1:**
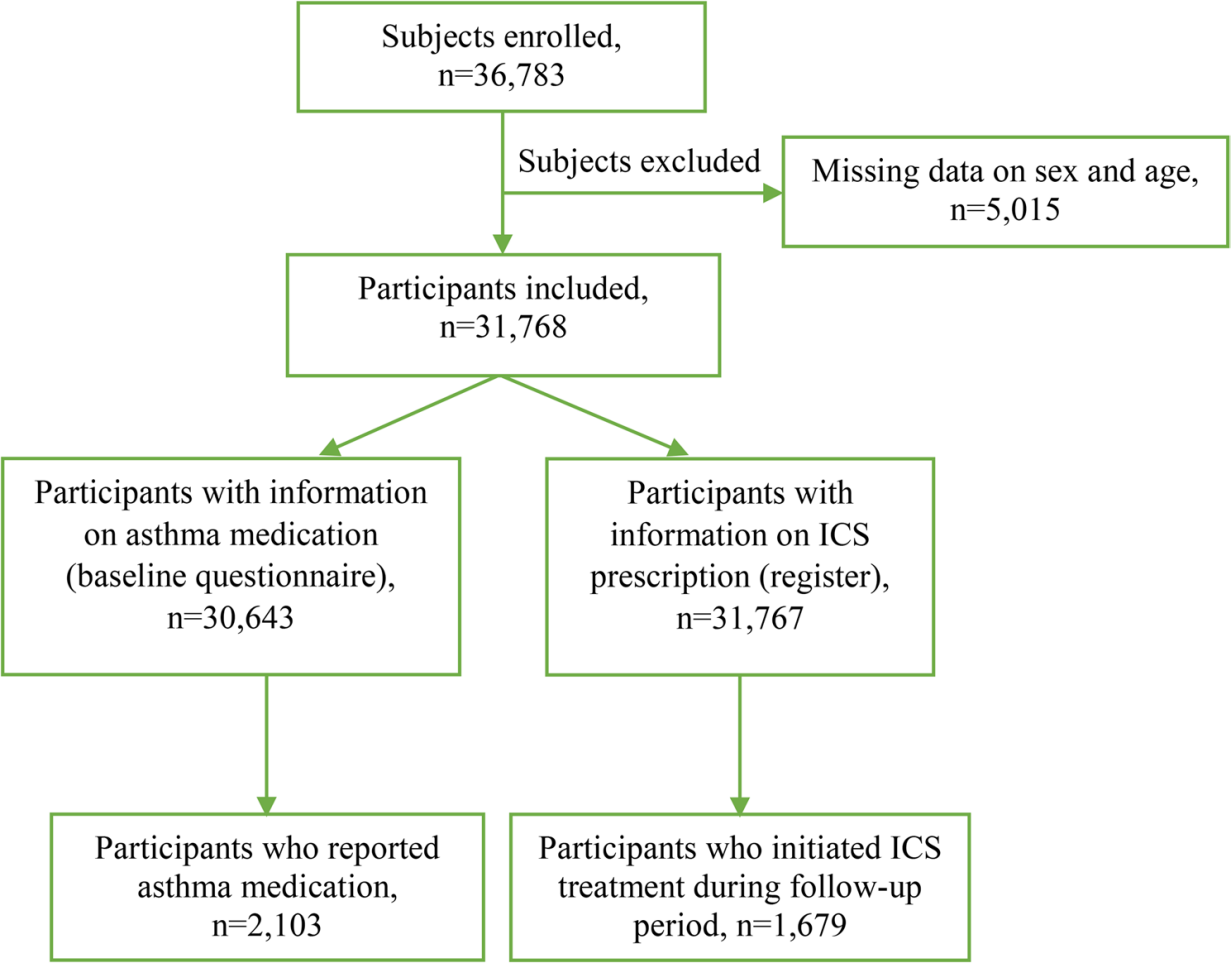
Flow chart of the studied population

Participants in the 20-year follow-up of the Respiratory Health in Northern Europe survey (RHINE III) in Sweden (*n* = 9,325) [9] and the Global Allergy and Asthma European Network (GA2LEN) survey in Sweden (*n* = 27,458) [27], both conducted in 2008–2010, are merged in the new cohort study REGAL. The new cohort includes participants from four Swedish cities: Stockholm, Gothenburg, Uppsala, and Umeå, and collected data on asthma diagnosis, asthma-related symptoms, home environment, and occupational exposures via questionnaires. The questionnaires used in RHINE III [28] and GA2LEN [29–31] have been published previously. Prescription of ICS from 2005 to 2018 was obtained from the Swedish National Prescribed Drug Registry, containing data on all dispensed prescriptions since 2005. The present analysis included 31,768 participants with valid information on sex and age from the REGAL database, of whom 26,571 were from the GA2LEN study. The study design is shown in Fig. 1.

### Assessment of demographics

Data on sex, age, height, weight, smoking history (never smoker/ex-smoker/current smoker), and educational level (primary school/secondary school/university or higher) were obtained through the questionnaires. Body mass index (BMI) was calculated by dividing weight by the square of height.

Population density was defined as inhabitants/km^2^ in each participant’s home area, based on Statistics Sweden’s demographic statistical areas [32]. Household size (number of persons living at home) was obtained from the population register at Statistics Sweden.

### Assessment of asthma by questionnaire

The questionnaire included a yes/no question about asthma medication: “Are you currently taking any asthma medication, including inhalers, aerosols, or tablets?”

The following asthma-related symptoms were asked for: wheezing or whistling in the chest at any time in the last 12 months; waking up with a feeling of tightness in the chest at any time in the last 12 months; waking up due to an attack of shortness of breath at any time in the last 12 months; and waking up due to an attack of coughing at any time in the last 12 months. “Current asthma-related symptom” was defined as the reporting of any of these four asthma-related symptoms.

### Register-based prescription of ICS

Information on prescription of ICS containing inhalers was obtained from the Swedish National Prescribed Drug Register from 2005 to 2018. Participants filling prescriptions for ICS containing inhalers were identified by ATC codes R03AK, R03BA, R03AL08, R03AL09, R03AL11, and R03AL12. Participants were considered to have started use of these inhalers if they filled at least two prescriptions within 12 months, and the date of filling the second ICS prescription was used as the start date of ICS treatment. From the date of questionnaire and date of ICS treatment start, participants initiating ICS treatment after answering questionnaires could therefore be identified (thereby initiating ICS treatment after reporting exposures). Participants who initiated or were on ICS treatment before the date of questionnaire were excluded. By requiring at least two ICS prescriptions, we aimed to include participants who had truly initiated and intended to maintain ICS therapy. Individuals with only one prescription were excluded because a single ICS prescription may reflect short-term treatment for an acute airway infection rather than chronic use.

### Assessment of environmental exposures in both RHINE and GA2LEN

Information on dampness and mould at home and traffic exposure at home were collected via questionnaires.

Participants were asked to report if they had noticed any of the following signs of dampness and mould at home during the last 12 months: (1) water damage, defined as water leakage or water damage indoors in walls, floor or ceilings; (2) floor dampness, defined as bubbles or yellow discoloration on plastic floor covering, or black discoloration of parquet floor; (3) visible mould, defined as visible mould growth indoors on walls, floor or ceilings.

Traffic exposure at home was assessed by two questions. The first question was asked in the RHINE questionnaire: “Do you hear traffic noise in your bedroom?”: (1) not at all, (2) a little, (3) much, and (4) very much; alternatives (3) and (4) were defined as “having troublesome traffic near home”. The second question was asked in the GA2LEN questionnaire: “How annoyed are you by fumes from traffic in your residential area?”: (1) none/a little (none), (2) somewhat and (3) very much, where reporting (3) was defined as “having troublesome traffic near home”.

### Assessment of environmental exposures only in GA2LEN

Information on irritating air at home, cleaning-related work, and previous occupational exposure to gas, smoke or dust were collected via questionnaire. Irritating air at home was defined as often finding the air in the residential area irritating. Participants could choose from three options: (1) daily/almost daily, (2) sometimes, and (3) seldom/never. Responses (1) and (2) were classified as affirmative for “irritating air at home”. Cleaning-related work was defined as currently working in a job that mainly involves some type of cleaning. Previous occupational exposure to gas, smoke or dust was defined as ever had a job being exposed to gas, smoke, or dust.

### Statistical analysis

Statistical analyses were performed using STATA 18. Categorical variables were summarised as percentages, while continuous variables were expressed as mean ± standard deviation (SD). Differences between participants with and without self-reported asthma medication at baseline, and between those who initiated and did not initiate ICS treatment during follow-up period, were assessed using Chi-square tests for categorical variables and t-tests for continuous variables. Pearson correlation analyses between environmental exposure variables were performed. Logistic regression models were used to examine the associations between environmental exposures and both self-reported asthma medication at baseline and initiating ICS treatment during follow-up period. As the next step, multiple logistic regression models were used to examine these associations, adjusting for five covariates: sex, age, BMI, smoking history, and educational level. Additional adjustment of household size and population density was also performed in these models. Similar analyses were performed among participants reporting any asthma-related symptoms at baseline, with adjustments for the five covariates. Sensitivity analyses were performed, restricting to never-smokers, and never-smokers in combination with age < 50 years. Kaplan-Meier failure estimates were conducted to show the relationship between exposure to environmental factors and time until initiating ICS treatment. The initiation of ICS treatment was defined as the first occurrence of two filled ICS prescriptions, where the second filled prescription was less than 12 months after the first filled prescription. Participants were followed for 10 years and continuously assessed for meeting this criterion. Thus, the cumulative hazard curves reflect the timing of the initiation of ICS treatment over the follow-up period. Additionally, the associations between environmental exposures and initiating ICS treatment were analysed using Cox proportional hazards regression models, adjusting for the aforementioned five covariates. Number Needed to Harm (NNH) was calculated based on the difference of risk (absolute risk difference) in exposed group and non-exposed group for relevant environmental exposures. The associations were expressed as odds ratios (OR) with a 95% confidence interval (CI) in logistic regression models, and hazard ratios (HR) with a 95% CI in Cox regression models, with statistical significance defined as *p* < 0.05.

## Results

A total of 31,768 subjects with valid data on sex and age were included in the present analysis (age 45.2 ± 15.4 years, 54.4% women) (Table 1). The RHINE and GA2LEN participants have similar baseline characteristics regarding sex, BMI, smoking habit and educational level, except that RHINE participants were older compared to GA2LEN participants (Table S1). The median follow-up time is 10.7 years. The prevalence of self-reported asthma medication use at baseline was 6.9% (*n* = 2,103), and 5.3% (*n* = 1,679) initiated ICS treatment during the follow-up period. However, 266 out of 1,679 who initiated ICS after baseline according to register reported that they were on asthma medication according to the baseline questionnaire, even though they had not prescribed ICS during the 3–5 years prior to answering the questionnaire. These 266 individuals were therefore included in the ICS initiation group for further analyses. Those initiating ICS treatment after the questionnaire survey were older, more likely to be women, had a higher BMI, were more often current smokers, and had a lower educational level. A similar trend was found among those with self-reported asthma medication. The median value of household size was 2 (ranging from 1 to 16), and the median value of population density was 2,727 inhabitants/km^2^ (ranged from 0.18 to 56,681 inhabitants/km^2^).

**Table 1:** Demographic characteristics of the participants in REGAL (*n* = 31,768)

		Total (*n* = 31,768)	Asthma medication (baseline questionnaire) (*n* = 30,643)		*p*	Initiation of ICS treatment during follow-up period (register) (*n* = 31,767)		*p*
			No (*n* = 28,540)	Yes (*n* = 2,103)		No (*n* = 30,088)	Yes (*n* = 1,679)	
Age (y)	Mean ± SD ^a^	45.2 ± 15.4	45.1 ± 15.3	44.5 ± 15.2	0.056	45.0 ± 15.4	48.1 ± 14.7	**< 0.001**
Women	% (n) ^b^	54.4 (17,291)	53.8 (15,354)	60.0 (1,261)	**< 0.001**	54.0 (16,240)	62.5 (1,050)	**< 0.001**
BMI (kg/m^2^)	Mean ± SD ^a^	25.0 ± 4.2	24.9 ± 4.2	25.9 ± 5.0	**< 0.001**	24.9 ± 4.2	25.8 ± 4.6	**< 0.001**
Smoking history	Never	59.7 (18,742)	60.0 (16,987)	58.2 (1,215)	**0.025**	60.1 (17,849)	53.4 (893)	**< 0.001**
	Ex-smoker	26.5 (8,331)	26.3 (7,440)	28.9 (604)		26.3 (7,820)	30.5 (510)	
	Current smoker	13.8 (4,326)	13.7 (3,886)	12.8 (268)		13.7 (4,057)	16.1 (269)	
Education level	Primary school	15.6 (4,896)	15.1 (4,272)	17.0 (354)	**0.002**	15.4 (4,581)	18.9 (315)	**< 0.001**
	Secondary school	35.2 (11,074)	35.1 (9,933)	37.2 (776)		35.4 (10,531)	32.5 (542)	
	University or higher	49.2 (15,473)	49.9 (14,120)	45.9 (959)		49.2 (14,662)	48.6 (811)	

Bold values indicate *p* < 0.05

^a^ t-test. SD means standard deviation

^b^ Chi-square test

The prevalence of water damage, floor dampness, and visible mould within the past 12 months was 7.1%, 3.8%, and 3.9%, respectively. A total of 17.9% of participants reported exposure to irritating air at home. Traffic-related exposure at home was reported by 4.0%. In addition, 36.7% reported previous occupational exposure to gas, smoke, or dust (Table 2). Pearson correlation analyses showed that all correlation coefficients were low (*r* < 0.4) (Table S2).

**Table 2:** Home environment factors in relation to self-reported asthma medication at baseline

		Prevalence of exposure (%)	Asthma medication (baseline questionnaire) (*n* = 30,643)			
			OR (95% CI) ^a^	*p*	OR (95% CI) ^b^	*p*
REGAL exposures						
Water damage in the last 12 months	Yes	7.1%	1.02 (0.86–1.21)	0.846	0.97 (0.81–1.16)	0.748
Floor dampness in the last 12 months	Yes	3.8%	1.41 (1.15–1.74)	**0.001**	1.32 (1.06–1.63)	**0.012**
Visible mould in the last 12 months	Yes	3.9%	0.86 (0.67–1.11)	0.248	0.81 (0.62–1.05)	0.108
Traffic exposure at home	Yes	4.0%	1.52 (1.25–1.85)	**< 0.001**	1.48 (1.21–1.81)	**< 0.001**
GA2LEN exposures						
Irritating air at home	Daily or sometimes	17.9%	2.21 (1.98–2.46)	**< 0.001**	2.08 (1.85–2.33)	**< 0.001**
Cleaning-related work	Yes	7.7%	1.05 (0.86–1.28)	0.628	0.90 (0.73–1.10)	0.308
Previous occupational exposure to gas, smoke or dust	Yes	36.7%	1.46 (1.32–1.61)	**0.001**	1.55 (1.39–1.72)	**< 0.001**

Bold values indicate *p* < 0.05

^a^ Logistic regression models, unadjusted

^b^ Logistic regression models adjusting for sex, age, BMI, smoking history and educational level

### Self-reported asthma medication at baseline

Floor dampness, irritating air at home, and traffic exposure at home were associated with self-reported asthma medication at baseline (Table 2). Moreover, reporting previous occupational exposure to gas, smoke, or dust was associated with self-reported asthma medication at baseline. Cleaning-related work was not significantly related to self-reported asthma medication. These associations remained significant in models adjusted for sex, age, BMI, smoking history, and educational level (Table 2), as well as in models with additional adjustments for household size and population density (Table S3).

### Future initiation of ICS during follow-up period

Reporting floor dampness and irritating air at home were associated with initiation of ICS treatment during follow-up period (Table 3). Moreover, previous occupational exposure to gas, smoke, or dust was associated with initiating ICS treatment. However, the associations between traffic exposure at home, cleaning-related work and initiation of ICS treatment were not significant. After adjusting for sex, age, BMI, smoking history, and educational level, these associations remained significant (Table 3), as well as in models with additional adjustments for household size and population density (Table S4). The associations remained in the sensitivity analyses restricted to never-smokers (Table S5). Similar associations were found when restricting to both never-smokers and age < 50 years, except that there was no association between floor dampness and initiation of ICS (*p* = 0.063) (Table S6).

**Table 3:** Home environment factors in relation to initiation of ICS treatment during follow-up period

		Initiation of ICS treatment during follow-up period (register) (*n* = 31,767)			
		OR (95% CI) ^a^	*p*	OR (95% CI) ^b^	*p*
REGAL exposures					
Water damage in the last 12 months	Yes	1.04 (0.86–1.26)	0.684	1.04 (0.86–1.26)	0.689
Floor dampness in the last 12 months	Yes	1.44 (1.15–1.81)	**0.001**	1.44 (1.15–1.82)	**0.002**
Visible mould in the last 12 months	Yes	0.92 (0.70–1.20)	0.517	0.94 (0.72–1.24)	0.686
Traffic exposure at home	Yes	1.21 (0.96–1.53)	0.111	1.14 (0.90–1.44)	0.268
GA2LEN exposures					
Irritating air at home	Daily or sometimes	1.51 (1.33–1.71)	**< 0.001**	1.47 (1.29–1.67)	**< 0.001**
Cleaning-related work	Yes	1.20 (0.98–1.46)	0.075	1.10 (0.90–1.36)	0.353
Previous occupational exposure to gas, smoke or dust	Yes	1.15 (1.03–1.28)	**0.015**	1.26 (1.12–1.41)	**< 0.001**

Bold values indicate *p* < 0.05

^a^ Logistic regression models, unadjusted

^b^ Logistic regression models adjusting for sex, age, BMI, smoking history and educational level

The NNH for exposures with significant associations to ICS initiation, was 47 for floor dampness at home, 42 for irritating air at home, and 141 for previous occupational exposure to gas, smoke, or dust. These values indicate that, over the 10-year follow-up, approximately 47, 42, and 141 individuals would need to be exposed to floor dampness, irritating air, and occupational gas/smoke/dust, respectively, for one additional person to initiate ICS treatment relative to the corresponding non-exposed group.

### Sub-group analyses among subjects with asthma symptoms at baseline

Among subjects with any asthma-related symptom at baseline, floor dampness was associated with initiating ICS treatment during follow-up period (Table 5). Visible mould was negatively associated with self-reported asthma medication at baseline (Table 4). Irritating air at home was associated with both self-reported asthma medication at baseline and initiating ICS treatment during follow-up period (Tables 4 and 5). Previous occupational exposure to gas, smoke, or dust was associated with self-reported asthma medication at baseline (Table 4).

**Table 4:** Home environment factors in relation to self-reported asthma medication at baseline, adjusting for covariates, among subjects reporting any asthma-related symptom at baseline

		Asthma medication (baseline questionnaire) (*n* = 11,316)	
		OR (95% CI) ^a^	*p*
REGAL exposures			
Water damage in the last 12 months	Yes	0.90 (0.74–1.09)	0.269
Floor dampness in the last 12 months	Yes	1.06 (0.84–1.34)	0.597
Visible mould in the last 12 months	Yes	0.71 (0.54–0.94)	**0.015**
Traffic exposure at home	Yes	1.23 (0.98–1.53)	0.071
GA2LEN exposures			
Irritating air at home	Daily or sometimes	1.51 (1.33–1.71)	**< 0.001**
Cleaning-related work	Yes	0.82 (0.65–1.01)	0.067
Previous occupational exposure to gas, smoke or dust	Yes	1.20 (1.06–1.35)	**0.003**

Bold values indicate *p* < 0.05

^a^ Logistic regression models adjusting for sex, age, BMI, smoking history and educational level

**Table 5:** Home environment factors in relation to initiation of ICS treatment during follow-up period, adjusting for covariates, among subjects reporting any asthma-related symptom at baseline

		Initiation of ICS treatment during follow-up period (register) (*n* = 11,773)	
		OR (95% CI) ^a^	*p*
REGAL exposures			
Water damage in the last 12 months	Yes	0.88 (0.69–1.13)	0.328
Floor dampness in the last 12 months	Yes	1.36 (1.05–1.78)	**0.022**
Visible mould in the last 12 months	Yes	0.77 (0.55–1.09)	0.142
Traffic exposure at home	Yes	0.94 (0.71–1.26)	0.696
GA2LEN exposures			
Irritating air at home	Daily or sometimes	1.19 (1.02–1.39)	**0.031**
Cleaning-related work	Yes	1.04 (0.81–1.34)	0.751
Previous occupational exposure to gas, smoke or dust	Yes	1.07 (1.92–1.24)	0.363

Bold values indicate *p* < 0.05

^a^ Logistic regression models adjusting for sex, age, BMI, smoking history and educational level

### The temporal relationship between exposures and initiation of ICS treatment during follow-up period

**Fig. 2:**
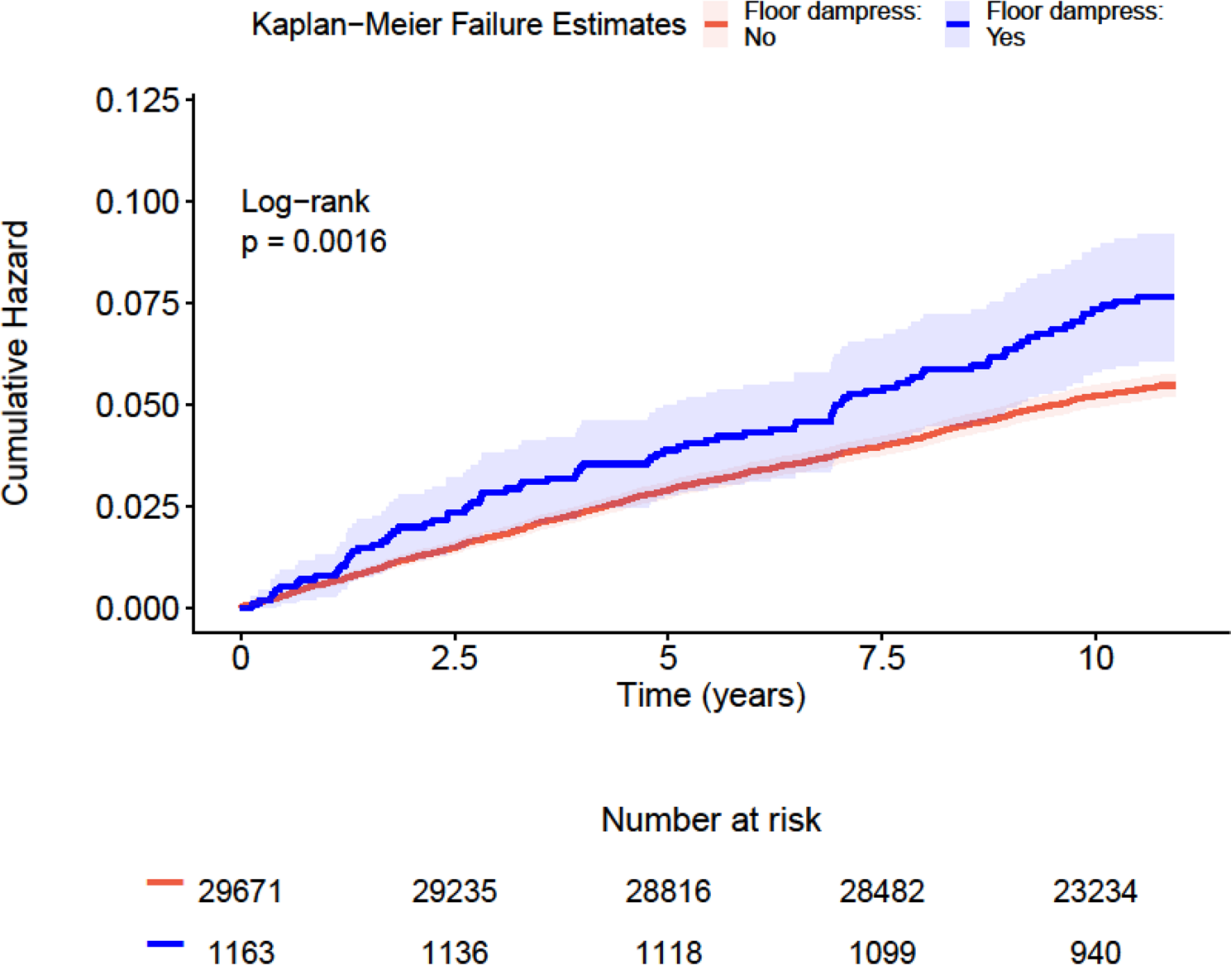
Kaplan–Meier graph showing floor dampness in relation to time until initiating ICS treatment

**Fig. 3:**
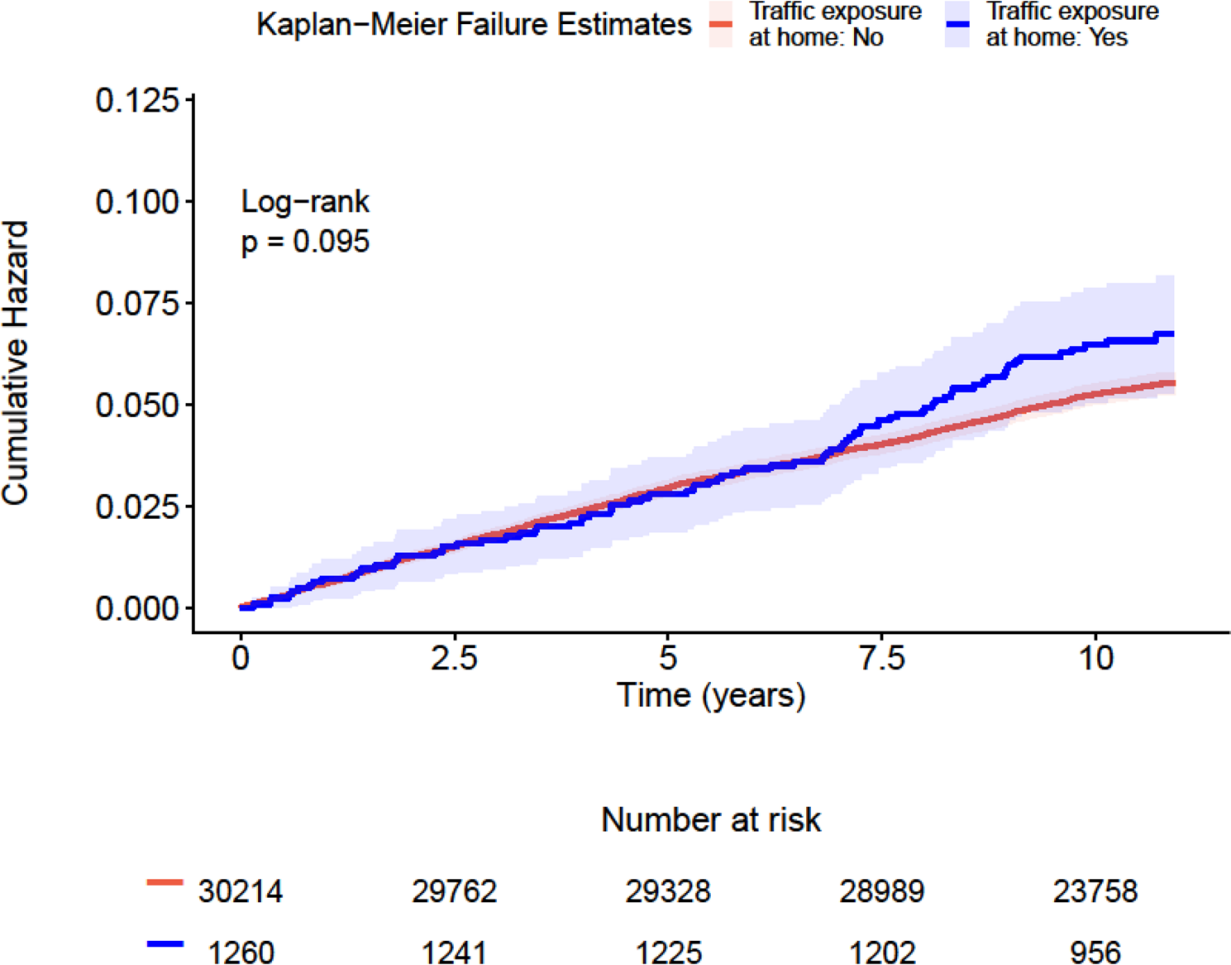
Kaplan–Meier graph showing traffic exposure at home in relation to time until initiating ICS treatment

**Fig. 4:**
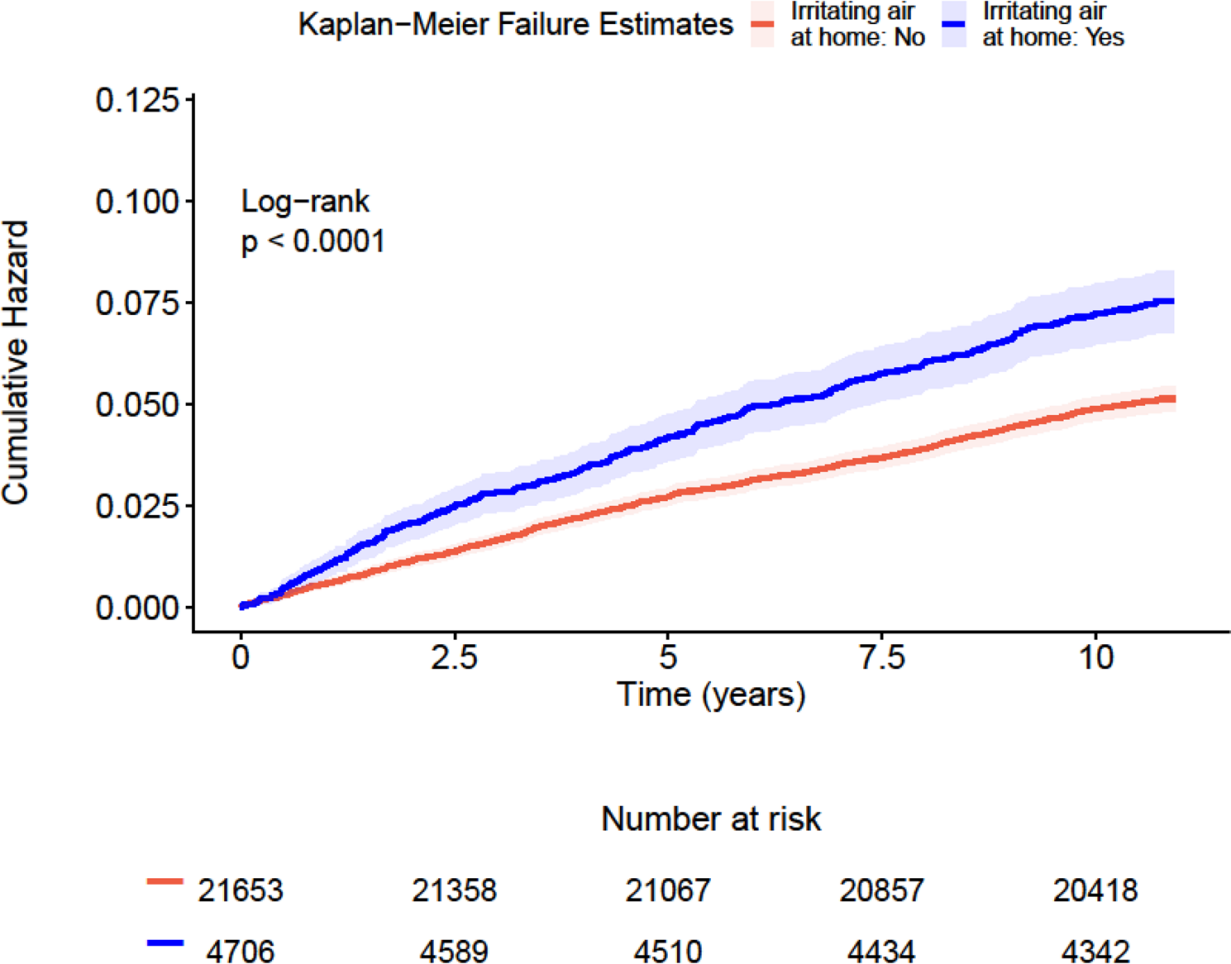
Kaplan–Meier graph showing irritating air at home in relation to time until initiating ICS treatment

**Fig. 5:**
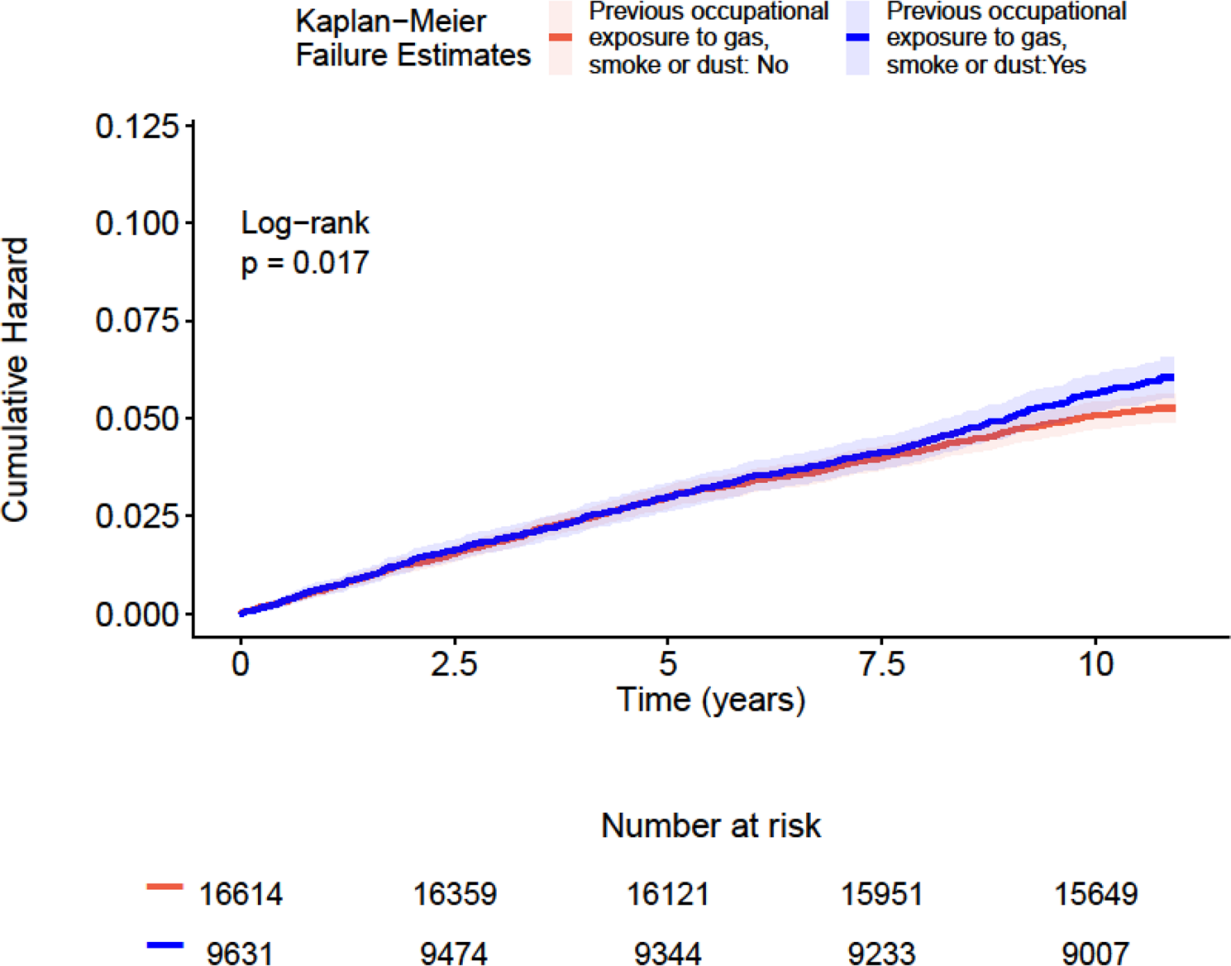
Kaplan–Meier graph showing previous occupational exposure to gas, smoke, or dust in relation to time until initiating ICS treatment

Floor dampness, traffic exposure at home, irritating air at home, and previous occupational exposure to gas, smoke, or dust in relation to time until initiating ICS treatment are shown in Kaplan-Meier graphs (Figs. 2, 3, 4 and 5). The unadjusted incidence of initiating ICS treatment was significantly higher among those exposed to floor dampness and irritating air at home. The incidence of initiating ICS treatment was also higher among those with previous occupational exposure to gas, smoke or dust, but this increase only became apparent after seven years of follow-up. Table 6 shows the hazard ratios from Cox proportional hazards regression models for these environmental exposures on initiating ICS treatment, adjusting for sex, age, BMI, smoking history and educational level. Floor dampness at home, irritating air at home, and previous occupational exposure to gas, smoke, or dust were associated with an increased risk of initiating ICS treatment during follow-up period.

**Table 6:** Risk of initiating ICS treatment during follow-up period from reported home environment factors

		Initiation of ICS treatment during follow-up period (register) (*n* = 31,767)	
		Hazard ratio (95% CI) ^a^	*p*
REGAL exposures			
Floor dampness in the last 12 months	Yes	1.42 (1.14–1.77)	**0.002**
Traffic exposure at home	Yes	1.14 (0.91–1.42)	0.271
GA2LEN exposures			
Irritating air at home	Daily or sometimes	1.46 (1.29–1.65)	**< 0.001**
Previous occupational exposure to gas, smoke or dust	Yes	1.25 (1.12–1.40)	**< 0.001**

Bold values indicate *p* < 0.05

^a^ Cox regression models adjusting for sex, age, BMI, smoking history and educational level

## Discussion

In this large population-based study, floor dampness, irritating air, and traffic exposure at home, as well as occupational exposure to gas, smoke, or dust, were associated with self-reported asthma medication. Moreover, floor dampness, irritating air, and occupational exposures to gas, smoke, or dust were linked to an increased risk of initiating ICS treatment in the future, where floor dampness and irritating air had the strongest associations. These associations persisted after adjustment for potential confounders. This indicates that these multiple exposures lead to a clinically meaningful respiratory disease.

Our study revealed a consistent association between floor dampness at home and both self-reported asthma medication use at baseline and subsequent initiation of ICS during follow-up period. This aligns with findings from two prospective European studies, which reported that household dampness and mould were linked to the development of asthma and respiratory symptoms in adults [8, 9]. Importantly, compared to water damage and visible mould, floor dampness showed the strongest association with incident doctor-diagnosed asthma in the long-term cohort [9], further supporting our results. A previous Swedish study suggested that dampness-related degradation of building materials caused emissions may explain the observed association between floor dampness and increased asthma symptoms in occupants [33]. A potential explanation for the negative association between visible mould and self-reported asthma medication at baseline in the subgroup with asthma-related symptoms could be that these participants were more prone to improving their home environment due to their symptoms.

We found that frequently perceived irritating air at home was associated with both self-reported asthma medication use at baseline and future initiation of ICS therapy. To our knowledge, no previous prospective studies have examined complaints of indoor air in the home environment in relation to new-onset asthma. However, a population-based cohort study from Northern Europe reported that self-reported mould odour at home showed the strongest association with new-onset asthma and nocturnal chest tightness [9]. A previous cross-sectional study from Sweden showed that complaints of stuffy air or unpleasant odour at home were associated with increased indoor air humidity, higher measured indoor moisture load, observed dampness in the foundation, and observed visible mould in the attic [34]. This suggests that perceived irritating air can be an indicator of impaired indoor environment, and may indicate a risk of developing respiratory disease.

In the present study, occupational exposures to gas, smoke, or dust were linked to an increased risk of medication use, including both self-reported asthma medication use at the time of the survey and initiating ICS in the future. This finding is in agreement with results from three recent population-based cohorts. The 10-year follow-up of the European Community Respiratory Health Survey (ECRHS) reported an increased risk of new-onset asthma among participants who experienced acute symptomatic inhalation events, such as exposure to fire, mixed cleaning agents, or chemical spills [21]. The 20-year follow-up of the ECRHS further demonstrated that exposure to wood dust was associated with a higher incidence of adult-onset asthma [22]. Similarly, a large population-based cohort study in Northern Europe found that exposure to irritant gases or fumes increased the risk of developing asthma in adults [23, 24]. A recent longitudinal study from Norway also reported that self-reported occupational exposure to vapours, gases, dust, and fumes was linked to a higher risk of new-onset asthma, particularly with frequent exposure [25]. Moreover, a study from the USA investigating workers in pulp and paper plants found that those who reported chronic or recurrent exposure to irritants had an increased risk of developing asthma [35]. Our results strengthened previous findings by showing the negative effects of hazardous occupational exposures and the future need for ICS treatment, although interestingly, this effect became most apparent seven years after the date of questionnaire (Fig. 5). This marks the long-term clinical relevance of these exposures. Also, the delayed effect highlights the cumulative risk of long-term exposures to poor indoor environment and supports the need for sustained preventive measures.

While self-reported traffic exposure at home was associated with self-reported asthma medication use, no significant association was found with future initiation of ICS therapy. This differs from previous prospective studies from Europe that have linked traffic-related particle matter [14, 16, 36], NO_2_ [15, 16] and black carbon [16] and increased risk of asthma onset in adults. Similarly, no significant association was observed between cleaning-related work and either self-reported asthma medication or prescribed ICS treatment in our study. This finding is consistent with the 10-year follow-up of the ECRHS, which reported no association between cleaning or caretaking and asthma incidence [21], and with a German cohort study that found no link between long-term use of disinfectants or cleaning products and asthma onset in young adults [26]. These results contrast with other prospective studies reporting an increased risk of asthma onset associated with occupational cleaning agents [22–24, 37]. The absence of such associations in our study may be explained by lower traffic-related exposure in Sweden, and lower exposure intensity or variability in cleaning tasks among the study population.

This study included more than 30,000 participants, providing a strong sample size that increases statistical power and allows for the detection of even modest effects. Participants were originally recruited from the RHINE and GA2LEN studies and were randomly selected from the general population across a broad geographical area in Sweden. This broad sampling frame strengthens the generalisability of the findings to the Swedish adult population. The study combined subjective data—collected via questionnaires—on environmental exposures, along with both subjective (questionnaire-based) and objective data (from the Swedish National Prescribed Drug Register) on medication use, to explore potential associations between environmental exposure and asthma in adults. The original response rates in the RHINE and GA²LEN studies were relatively high (RHINE III 62%, GA2LEN 60%) [9, 38, 39], further supporting the validity of the sample. Our study may be susceptible to Type I error due to multiple testing; however, the consistent patterns observed across models strengthen confidence in the robustness of our findings. The main limitations are the absence of clinical measurements for asthma diagnosis, and that all environmental exposure information was self-reported. The cross-sectional study design used to assess environmental exposures in relation to self-reported asthma medication limited the ability to draw causal inferences. However, the ability to link baseline exposure data with registry data on future prescriptions of ICS is a key strength of our study.

## Conclusions

In this large population-based study, indoor dampness, perceived irritating air at home, and occupational environmental exposures to gas, smoke, or dust were associated with self-reported asthma medication, and were also predictive of subsequent initiation of ICS therapy. This highlights the need for better public health interventions targeting improvements in indoor environments. Identifying and reducing environmental exposures at home and in the workplace may help prevent or manage asthma in adults.

## Supplementary Information

Supplementary Material 1.

## Data Availability

Data cannot be made freely available as they are subject to secrecy in accordance with the Swedish Public Access to Information and Secrecy Act, but can be made available to researchers upon request, after approval from the Swedish Ethical Review Authority has been obtained. Please contact Össur Ingi Emilsson (email: ossur.emilsson@medsci.uu.se) to obtain access to the raw data used in our study.

## Abbreviations

BMI: Body mass index
CI: Confidence interval
ECRHS: European Community Respiratory Health Survey
GA2LEN: Global Allergy and Asthma European Network
ICS: Inhaled corticosteroid
NO2: Nitrogen dioxide
RHINE: Respiratory Health in Northern Europe survey
REGAL: The combined cohort based on the Swedish part of the Respiratory Health in Northern Europe survey and the Swedish part of the Global Allergy and Asthma European Network survey
SD: Standard deviation
