# Supplementary material for: Environmental exposures at home and in the workplace in relation to inhaled corticosteroid medication - the population-based REGAL study
Source: BMC Pulm Med. 2026 Jul 20;26:321. doi: 10.1186/s12890-026-04502-w (PMC13383413; doi:10.1186/s12890-026-04502-w)
Supplement: Supplementary file 1 — Supplementary Material 1. [file 12890_2026_4502_MOESM1_ESM.docx]

**Supplement**

Table S1. Demographic characteristics of the participants in RHINE III and GA2LEN.

|  |  | Total  (n=31,768) | RHINE III  (n=5,425) | GA2LEN  (n=26,343) |
| --- | --- | --- | --- | --- |
| Age (y) | Mean±SD ^a^ | 45.2±15.4 | 52.3±7.3 | 43.7±16.2 |
| Women | % (n) | 54.4 (17,291) | 53.7 (2,912) | 54.6 (14,379) |
| BMI (kg/m^2^) | Mean±SD ^a^ | 25.0±4.2 | 25.9±4.2 | 24.7±4.2 |
| Smoking history | Never | 59.7 (18,742) | 54.1 (2,878) | 60.8 (15,864) |
|  | Ex-smoker | 26.5 (8,331) | 32.7 (1,736) | 25.3 (6,595) |
|  | Current smoker | 13.8 (4,326) | 13.2 (703) | 13.9 (3,623) |
| Education level | Primary school | 15.6 (4,896) | 13.0 (705) | 16.1 (4,191) |
|  | Secondary school | 35.2 (11,074) | 41.7 (2,258) | 33.9 (8,816) |
|  | University or higher | 49.2 (15,473) | 45.3 (2,451) | 50.0 (13,022) |

^a^ SD means standard deviation.

Table S2. Pearson correlation coefficients for home environment variables.

|  |  |  |  |  |
| --- | --- | --- | --- | --- |
| **REGAL exposures** | Water damage in the last 12 months | Floor dampness in the last 12 months | Visible mould in the last 12 months | Traffic exposure in the last 12 months |
| Water damage in the last 12 months | 1.00 |  |  |  |
| Floor dampness in the last 12 months | 0.29 | 1.00 |  |  |
| Visible mould in the last 12 months | 0.36 | 0.24 | 1.00 |  |
| Traffic exposure at home | 0.03 | 0.05 | 0.04 | 1.00 |
| **GA2LEN exposures** | Irritating air at home | Cleaning-related work | Previous occupational exposure to gas, smoke or dust |  |
| Irritating air at home | 1.00 |  |  |  |
| Cleaning-related work | 0.08 | 1.00 |  |  |
| Previous occupational exposure to gas, smoke or dust | 0.05 | 0.11 | 1.00 |  |

Table S3. Home environment factors in relation to self-reported asthma medication at baseline, adjusting for covariates.

|  |  | Asthma medication (baseline questionnaire) (n=30,643) | |
| --- | --- | --- | --- |
|  |  | OR (95% CI) ^a^ | p |
| **REGAL exposures** |  |  |  |
| Water damage in the last 12 months | Yes | 0.97 (0.81-1.17) | 0.769 |
| Floor dampness in the last 12 months | Yes | 1.30 (1.05-1.61) | **0.017** |
| Visible mould in the last 12 months | Yes | 0.82 (0.63-1.06) | 0.129 |
| Traffic exposure at home | Yes | 1.55 (1.26-1.89) | **<0.001** |
| **GA2LEN exposures** |  |  |  |
| Irritating air at home | Daily or sometimes | 2.14 (1.91-2.41) | **<0.001** |
| Cleaning-related work | Yes | 0.93 (0.76-1.14) | 0.491 |
| Previous occupational exposure to gas, smoke or dust | Yes | 1.54 (1.38-1.72) | **<0.001** |

Bold values indicate p<0.05.

^a^ Logistic regression models adjusting for sex, age, BMI, smoking history, educational level, household size, and population density.

Table S4. Home environment factors in relation to initiation of ICS treatment during follow-up period, adjusting for covariates.

|  |  | Initiation of ICS treatment during follow-up period (register) (n=31,767) | |
| --- | --- | --- | --- |
|  |  | OR (95% CI) ^a^ | p |
| **REGAL exposures** |  |  |  |
| Water damage in the last 12 months | Yes | 1.02 (0.84-1.24) | 0.825 |
| Floor dampness in the last 12 months | Yes | 1.44 (1.14-1.81) | **0.002** |
| Visible mould in the last 12 months | Yes | 0.92 (0.70-1.22) | 0.575 |
| Traffic exposure at home | Yes | 1.14 (0.90-1.44) | 0.283 |
| **GA2LEN exposures** |  |  |  |
| Irritating air at home | Daily or sometimes | 1.49 (1.31-1.70) | **<0.001** |
| Cleaning-related work | Yes | 1.10 (0.90-1.36) | 0.357 |
| Previous occupational exposure to gas, smoke or dust | Yes | 1.26 (1.12-1.42) | **<0.001** |

Bold values indicate p<0.05.

^a^ Logistic regression models adjusting for sex, age, BMI, smoking history, educational level, household size, and population density.

Table S5. Home environment factors in relation to initiation of ICS treatment during follow-up period, adjusting for covariates, among never-smokers (n=18,742).

|  |  | Initiation of ICS treatment during follow-up period (register) | |
| --- | --- | --- | --- |
|  |  | OR (95% CI) ^a^ | p |
| **REGAL exposures** |  |  |  |
| Water damage in the last 12 months | Yes | 0.98 (0.74-1.28) | 0.863 |
| Floor dampness in the last 12 months | Yes | 1.58 (1.17-2.15) | **0.003** |
| Visible mould in the last 12 months | Yes | 0.68 (0.44-1.05) | 0.083 |
| Traffic exposure at home | Yes | 1.04 (0.73-1.49) | 0.816 |
| **GA2LEN exposures** |  |  |  |
| Irritating air at home | Daily or sometimes | 1.48 (1.24-1.76) | **<0.001** |
| Cleaning-related work | Yes | 1.10 (0.82-1.48) | 0.508 |
| Previous occupational exposure to gas, smoke or dust | Yes | 1.35 (1.15-1.57) | **<0.001** |

Bold values indicate p<0.05.

^a^ Logistic regression models adjusting for sex, age, BMI, smoking history, and educational level.

Table S6. Home environment factors in relation to initiation of ICS treatment during follow-up period, adjusting for covariates, among never-smokers and age<50 years (n=12,871).

|  |  | Initiation of ICS treatment during follow-up period (register) | |
| --- | --- | --- | --- |
|  |  | OR (95% CI) ^a^ | p |
| **REGAL exposures** |  |  |  |
| Water damage in the last 12 months | Yes | 0.85 (0.61-1.19) | 0.348 |
| Floor dampness in the last 12 months | Yes | 1.41 (0.98-2.02) | 0.063 |
| Visible mould in the last 12 months | Yes | 0.65 (0.40-1.07) | 0.091 |
| Traffic exposure at home | Yes | 0.95 (0.59-1.52) | 0.825 |
| **GA2LEN exposures** |  |  |  |
| Irritating air at home | Daily or sometimes | 1.38 (1.12-1.70) | **0.003** |
| Cleaning-related work | Yes | 1.13 (0.81-1.59) | 0.480 |
| Previous occupational exposure to gas, smoke or dust | Yes | 1.32 (1.10-1.59) | **0.003** |

Bold values indicate p<0.05.

^a^ Logistic regression models adjusting for sex, age, BMI, smoking history, and educational level.
